# Double-strand break repair and colorectal cancer: gene variants within 3′ UTRs and microRNAs binding as modulators of cancer risk and clinical outcome

**DOI:** 10.18632/oncotarget.6804

**Published:** 2015-12-31

**Authors:** Alessio Naccarati, Fabio Rosa, Veronika Vymetalkova, Elisa Barone, Katerina Jiraskova, Cornelia Di Gaetano, Jan Novotny, Miroslav Levy, Ludmila Vodickova, Federica Gemignani, Tomas Buchler, Stefano Landi, Pavel Vodicka, Barbara Pardini

**Affiliations:** ^1^ Molecular and Genetic Epidemiology Research Unit, Human Genetics Foundation, Turin, Italy; ^2^ Department of Molecular Biology of Cancer, Institute of Experimental Medicine, Prague, Czech Republic; ^3^ Genomic Variation in Human Populations and Complex Diseases Research Unit, Human Genetics Foundation, Turin, Italy; ^4^ Institute of Biology and Medical Genetics, First Faculty of Medicine, Charles University, Prague, Czech Republic; ^5^ Department of Biology, University of Pisa, Pisa, Italy; ^6^ Department of Medical Sciences, University of Turin, Turin, Italy; ^7^ Department of Oncology, First Faculty of Medicine, Charles University, Prague, Czech Republic; ^8^ Department of Surgery, First Faculty of Medicine, Charles University and Thomayer University Hospital, Prague, Czech Republic; ^9^ Department of Oncology, Thomayer Hospital and First Faculty of Medicine, Charles University, Prague, Czech Republic

**Keywords:** double-strand break repair (DSBR) genes, colorectal cancer risk and clinical outcomes, miRNA binding sites, 3′UTR polymorphisms, MRE11A

## Abstract

Genetic variations in 3′ untranslated regions of target genes may affect microRNA binding, resulting in differential protein expression. microRNAs regulate DNA repair, and single-nucleotide polymorphisms in miRNA binding sites (miRSNPs) may account for interindividual differences in the DNA repair capacity. Our hypothesis is that miRSNPs in relevant DNA repair genes may ultimately affect cancer susceptibility and impact prognosis.

In the present study, we analysed the association of polymorphisms in predicted microRNA target sites of double-strand breaks (DSBs) repair genes with colorectal cancer (CRC) risk and clinical outcome. Twenty-one miRSNPs in non-homologous end-joining and homologous recombination pathways were assessed in 1111 cases and 1469 controls. The variant CC genotype of rs2155209 in *MRE11A* was strongly associated with decreased cancer risk when compared with the other genotypes (OR 0.54, 95% CI 0.38–0.76, *p* = 0.0004). A reduced expression of the reporter gene was observed for the C allele of this polymorphism by *in vitro* assay, suggesting a more efficient interaction with potentially binding miRNAs. In colon cancer patients, the rs2155209 CC genotype was associated with shorter survival while the TT genotype of *RAD52* rs11226 with longer survival when both compared with their respective more frequent genotypes (HR 1.63, 95% CI 1.06-2.51, *p* = 0.03 HR 0.60, 95% CI 0.41–0.89, *p* = 0.01, respectively).

miRSNPs in DSB repair genes involved in the maintenance of genomic stability may have a role on CRC susceptibility and clinical outcome.

## INTRODUCTION

Colorectal cancer (CRC) is among the most frequent malignancies worldwide and is the third highest cause of cancer mortality among men and women [1]. Though CRC detected at an early stage can be successfully removed, cancers undetected until an advanced stage with metastases remain incurable [2]. The growing incidence of CRC (2001–2011 growth index 6.0%) was accompanied by a relatively low rate of early detection of the disease [3]. Therefore, there is an urgent need to find biomarkers to aid prevention, treatment and prognosis in CRC.

The molecular etiology of CRC has been explored extensively, revealing that this cancer develops from an accumulation of genomic mutations. Accumulating cellular DNA damage, if not correctly repaired, can lead to genomic instability, apoptosis or senescence and may ultimately predispose the organism to various disorders including cancers. The importance of DNA repair is highlighted by the fact that mutations in a number of DNA repair genes lead to human syndromes that include multiple cancers, immunodeficiency, and phenotypes with chromosomal anomalies [4]. There is a large body of evidence on the associations between DNA repair and the risk of cancer, including CRC [5].

The repair of double-strand breaks (DSBs), the most deleterious type of DNA damage, is a fundamental cellular mechanism to preserve genomic stability [6]. Two pathways are specifically dedicated to the repair of DSBs: homologous recombination (HRe) and non-homologous end joining (NHEJ) [7–9]. The repression of these efficient repair systems permits an accumulation of damage in rapidly dividing cells (such as cancer cells) that can induce apoptosis. Such an effect may also be exerted by radiation therapy (an inducer of DSBs) in cancer patients [7, 9, 10].

DNA repair capacity varies markedly among individuals, and there is evidence that its decrease is associated with increased cancer risk [11, 12]. In this respect, DNA repair genes present numerous single nucleotide polymorphisms (SNPs) with different allelic distributions in the general population. Some of these SNPs have been reported to be associated with cancer susceptibility in a number of malignancies that include CRC [13]. We have previously investigated associations between functional SNPs in DNA repair genes (including DSB repair genes *XRCC3* and *NBS1*) and CRC susceptibility in cases and controls from the Czech Republic [14, 15]. Our findings have suggested that variations in DNA repair genes may be associated with cancer susceptibility through an altered repair function that can also explain some of the phenotypic differences observed in CRC [11, 16, 17].

In recent years, there has been a growing interest in the role of post-transcriptional regulation of gene expression modulated by microRNAs (miRNAs). In concomitance, the importance of the SNPs located within miRNA-binding target sites (miRSNPs) on cancer risk has been highlighted [18, 19]. Regulation and coordination between genes involved in the DNA repair pathways are fundamental for maintaining genome stability, and post-transcriptional gene regulation by miRNAs is one of the critical players in these processes [20]. Thus, subtle effects displayed by SNPs in DNA repair signaling genes may account for some of these variations. In this sense, specific polymorphisms in regulatory regions such as miRNA target sites may also modulate survival and response to therapy in cancer patients [18, 21].

We recently reported associations between miRSNPs in genes of 3 DNA repair pathways (Nucleotide Excision Repair, Base Excision Repair and Mismatch Repair) and CRC risk or clinical outcome [21–23]. SNPs in miRNA target regions of important genes for DSBs repair may also affect the efficiency of translation of corresponding proteins. Thus, in the present study, we hypothesized that variations in DSB genes may modulate signaling response and the maintenance of genomic stability ultimately affecting cancer susceptibility, cancer survival and efficacy of chemotherapy. We investigated the role of 21 polymorphisms in miRNA predicted target sites of NHEJ and HRe genes in association with CRC risk and its clinical outcome in cases and controls from the Czech Republic.

## RESULTS

### miRSNP selection

Out of the 21 genes involved in the HRe pathway, only 11 had polymorphisms predicted to bind miRNAs in their 3′UTRs. After further selection based on MAF and LD study criteria (see Materials and Methods section), 15 miRSNPs within the 3′UTRs of seven genes (*RAD51*, *RAD52*, *BRCA1*, *MRE11A*, *NBN*, *GEN1* and *XRCC2*) were identified. For NHEJ, from the initial seven genes involved in the pathway, a total of 39 miRSNPs in the 3′UTRs were found. Since the majority of the SNPs are not represented in the Caucasian population, only six polymorphisms in four genes (*XRCC4*, *XRCC5*, *LIG4*, and *NHEJ1)* passing the selection criteria were finally included in the study.

### Case-control study

The characteristics of the study participants are presented in Table 1 [21].

**Table 1:** Characteristics of the study population

		Cases	Controls	OR	95% CI	*P*
Age (years)	[18, 47]	90	591	Ref		
	(47, 55]	208	422	3.24	2.45–4.27	< 0.00001
	(55, 65]	375	286	8.61	6.57–11.28	< 0.00001
	(65,91]	438	170	16.92	12.74–22.47	< 0.00001
Sex	Females	433	660	Ref		
	Males	678	809	1.28	1.09–1.50	0.003
BMI	[0, 23.7]	187	367	Ref		
	(23.7, 26.2]	195	362	1.06	0.82–1.35	0.70
	(26.2, 28.9]	229	323	1.39	1.09–1.78	0.01
	(28.9, 53.1]	224	329	1.34	1.05–1.71	0.02
Smoking	Non smokers	541	815	Ref		
	Smokers	161	328	0.74	0.59–0.92	0.006
	Ex-smokers	341	253	2.01	1.65–2.45	< 0.001
Family History CRC	No	736	1204	Ref		
	Yes	146	142	1.68	1.31–2.16	< 0.0001
Living Area	Town	520	952	Ref		
	Town and country	128	171	1.37	1.06–1.76	0.02
	Country	244	269	1.66	1.35–2.04	< 0.00001
Education	Primary	271	224	Ref		
	Secondary	473	819	0.48	0.39–0.59	< 0.00001
	University or higher	141	345	0.34	0.26–0.44	< 0.00001

Abbreviations: BMI: Body Mass Index OR, odds ratio, 95% CI, confidence interval

None of the 21 SNPs deviated from Hardy-Weinberg equilibrium in control subjects. The strongest association with CRC susceptibility was observed for rs2155209 in *MRE11A*, a gene involved in HRe. The variant genotype CC of this SNP was associated with a decreased risk of cancer (odds ratios (OR) 0.54, 95% confidence intervals (CI) 0.38–0.76, *p* = 0.0004). This association remained significant also after correction for multiple testing. Moreover, a similar significant association was observed when stratifying the case group according to tumor site (for rectal cancer: OR 0.32, 95% CI 0.18–0.59, *p* = 0.0002; for colon cancer: OR 0.66, 95% CI 0.45–0.96, *p* = 0.03) (Table 2 and Supplementary Table 1). Conversely, the variant genotype AA of *RAD52* rs1051669 was associated with increased risk of cancer (OR 1.68, 95% CI 1.11–2.54, *p* = 0.01).

**Table 2:** Significant associations of SNPs in HRe genes with CRC risk (stratification for colon and rectal cancer is also reported)

Gene SNP	Genotype	^a^Controls(*n* = 1442)	All cancer patients				Rectal cancer patients				Colon cancer patients			
			^a^Cases (*n* = 1090)	^b^OR	95% CI	*P*	^a^Cases (*n* = 369)	^b^OR	95% CI	*P*	Cases (*n* = 710)	^b^OR	95% CI	*P*
*RAD52*	GG	839	610	Ref			213	Ref			395	Ref		
rs1051669	GA	505	381	1.05	0.85–1.29	0.65	121	0.96	0.70–1.30	0.78	260	1.09	0.86–1.37	0.49
	AA	70	81	1.68	1.11–2.54	**0.01**	28	1.39	0.74–2.59	0.31	52	1.78	1.13–2.80	**0.01**
	GA+AA	575	462	1.12	0.92–1.37	0.25	149	1.01	0.75–1.35	0.97	312	1.17	0.93–1.46	0.18
	GG+GA	1344	991	Ref			334	Ref			655	Ref		
	AA	70	81	1.65	1.10–2.50	**0.02**	28	1.41	0.76–2.61	0.28	52	1.72	1.10–2.69	**0.02**
*RAD52*	TT	1045	820	Ref			266	Ref			551	Ref		
rs11571475	TC	353	237	0.88	0.70–1.11	0.29	88	1.14	0.82–1.59	0.45	149	0.76	0.58–1.00	**0.05**
	CC	24	20	0.90	0.41–1.96	0.79	12	2.36	0.97–5.73	0.06	8	0.41	0.14–1.26	0.12
	TC+CC	377	257	0.88	0.70–1.11	0.29	100	1.21	0.88–1.67	0.24	157	0.74	0.57–0.97	**0.03**
	TT+TC	1398	1057	Ref			354	Ref			700	Ref		
	CC	24	20	0.92	0.42–2.01	0.84	12	2.28	0.95–5.52	0.07	8	0.44	0.14–1.34	0.15
*RAD52*	TT	1024	808	Ref			280	Ref			526	Ref		
rs7963551	TG	375	246	0.83	0.66–1.04	0.11	80	0.88	0.64–1.23	0.47	165	0.81	0.63–1.05	0.12
	GG	35	17	0.53	0.26-1.11	0.09	5	0.29	0.08–1.13	0.07	12	0.64	0.29–1.38	0.25
	TG+GG	410	263	0.80	0.64–1.00	**0.05**	85	0.83	0.60–1.15	0.26	177	0.80	0.62–1.02	0.08
	TT+TG	1400	1054	Ref			360	Ref			691	Ref		
	GG	35	17	0.56	0.27–1.16	0.12	5	0.30	0.08–1.16	0.08	12	0.67	0.31–1.46	0.31
*MRE11A*	TT	610	499	Ref			181	Ref			316	Ref		
rs2155209	TC	638	485	0.86	0.70–1.06	0.16	162	0.75	0.56–1.01	0.06	322	0.94	0.75–1.19	0.61
	CC	180	92	0.54	0.38–0.76	**0.0004**	22	0.32	0.18–0.59	**0.0002**	70	0.66	0.45–0.96	**0.03**
	TC+CC	818	577	0.79	0.65–0.96	**0.02**	184	0.66	0.49–0.87	**0.004**	392	0.88	0.70–1.09	0.24
	TT+TC	1248	984	Ref			343	Ref			638	Ref		
	CC	180	92	0.58	0.42–0.81	**0.001**	22	0.37	0.21–0.67	**0.0009**	70	0.68	0.47–0.97	**0.03**
*NBN*	TT	1308	1010	Ref			343	Ref			664	Ref		
rs14448	TC	107	74	0.67	0.45–1.00	**0.05**	22	0.41	0.21–0.80	**0.01**	52	0.78	0.51–1.19	0.24
	CC	0	0	-	–	–	0	–	–	–	0	–	–	–
	TC+CC	107	74	0.67	0.45–1.00	**0.05**	22	0.41	0.21-0.80	**0.01**	52	0.78	0.51-1.19	0.24
	TT+TC	1415	1084	Ref			365	Ref			716	Ref		
	CC	0	0	–	–	–	0	–	–	–	0	–	–	–

Abbreviations: OR, odds ratio; 95% CI, confidence interval. Significant results in bold.

a Numbers may not add up to 100% of available subjects because of genotyping failure. All samples that did not give a reliable result in the first round of genotyping were resubmitted to up to two additional rounds of genotyping. Data points that were still not filled after this procedure had been left blank.

b Adjusted for sex, age and smoking.

c χ^2^ and *P*-values for the deviation of observed and the numbers expected from the Hardy–Weinberg equilibrium (HWE) considering all controls.

After stratification for tumor site, two polymorphisms in *RAD52* gene (rs1051669 and rs11571475) were associated with colon cancer risk while one SNP in *NBN* (rs14448) was associated with rectal cancer risk. In particular, carriers of the AA genotype or the variant A allele in rs1051669 were at increased risk to develop cancer in the colon (OR 1.78, 95% CI 1.13–2.80, *p* = 0.01 and OR 1.72, 95% CI 1.10–2.692, *p* = 0.02, respectively); whereas carriers of the heterozygous TC genotype of rs11571475 were at decreased risk to develop colon cancer (OR 0.76, 95% CI 0.58–1.00, *p* = 0.05). This last observed association should be cautiously considered: in the dominant model the presence of the variant C allele was associated with a decreased risk of colon cancer (OR 0.74, 95% CI 0.57–0.97, *p* = 0.03). However, due to the low frequency of the CC genotype in our study group it was not possible to observe the same effect in the co-dominant model (Table 2). A decreased risk of rectal cancer was observed for carriers of the heterozygous genotype in rs14448 (OR 0.41, 95% CI 0.21–0.80, *p* = 0.01).

Globally, no significant associations with the risk of CRC were found for any of the studied polymorphisms in the NHEJ pathway. The only observed exception was for *XRCC5* rs1051677 when comparing only rectal cancer patients with controls (codominant model: OR 3.84, 95% CI 1.11–13.31, *p* = 0.03; recessive model: OR 3.75, 95% CI1.08–12.95, *p* = 0.04) (Supplementary Table 2).

### Contingency tables for SNP interaction analyses

As the variants under investigation are part of two DNA repair pathways where genes work functionally coupled, the polymorphisms emerging from the case-control study were also explored for their potential SNP-SNP interaction in modulating CRC susceptibility. In general, the results revealed a tendency for the under-representation of cases in comparison with controls among carriers of the variant rs2155209 genotype CC in *MRE11A* in combinations with other SNPs in genes of HRe pathway (Supplementary Table 3). Among the most interesting results, the observed protective effect of rs2155209 was increased by the concomitant presence of AA genotype of *XRCC2* rs3218547, whose protective effect was not reaching the significance when analysed alone. Conversely, there was an under-representation of *RAD52* rs1051669 AA genotype (alone associated with an increased risk to develop cancer) in carriers of the variant C allele of rs2155209 (Supplementary Table 3).

### Survival analyses

The mean (median) overall survival (OS) and event-free survival (EFS) for patients were 86.5 (80.5) and 72.6 (62.4) months, respectively. Age, gender, T, N, M status, chemotherapy treatment and CRC stage were associated with OS and EFS in the preliminary univariate assessment of covariates (Table 3). Advanced age, male gender and current smoking status were related to a shorter OS. Likewise, men were also at higher risk of relapse or metastasis (OS: Hazard ratio (HR) 1.54; 95% CI 1.23–1.92; *p* = 0.0001; EFS: HR 1.35; 95% CI 1.09–1.68; *p* = 0.006). Four established prognostic factors (T, N, M status and stage) were associated with decreased patients’ survival and increased risk of recurrence. Moreover, adjuvant chemotherapy was also associated with survival (Table 3).

**Table 3:** Clinical and anamnestic characteristics significantly affecting Overall Survival (OS) and Event Free Survival (EFS) of the CRC patients with complete follow up (Cox regression)

		OS			EFS	
		*N^a^*	HR (95% CI)	*P*	HR (95% CI)	*P*
Sex	Females	427	Ref		Ref	
	Males	656	1.54 (1.23–1.92)	**0.0001**	1.35 (1.09–1.68)	**0.006**
Age (years)	55 ≤	293	Ref		Ref	
	56–62	248	1.43 (1.05–1.95)	**0.02**	1.41 (1.06–1.87)	**0.02**
	63–70	294	1.39 (1.04–1.88)	**0.03**	1.19 (0.90–1.58)	0.22
	> 70	248	2.02 (1.50–2.72)	**< 0.0001**	1.04 (0.76–1.42)	0.80
Smoking habit^*^	No	533	Ref		Ref	
	Yes	493	1.26 (1.02–1.56)	**0.03**	1.14 (0.93–1.41)	0.19
pT	1	50	Ref		Ref	
	2	166	2.64 (0.94–7.40)	0.06	2.18 (0.85–5.55)	0.10
	3	535	5.84 (2.17–15.71)	**0.0005**	5.58 (2.30–13.53)	**0.0001**
	4	136	9.21 (3.36–25.26)	**< 0.0001**	6.96 (2.80–17.27)	**< 0.0001**
pN	0	498	Ref		Ref	
	1	260	2.17 (1.69–2.79)	**< 0.0001**	1.87 (1.46–2.41)	**< 0.0001**
	2	68	3.40 (2.35–4.91)	**< 0.0001**	3.43 (2.45–4.81)	**< 0.0001**
pM	0	725	Ref		Ref	
	1	177	4.80 (3.83–6.02)	**< 0.0001**	4.56 (3.68–5.65)	**< 0.0001**
5FU-based chemotherapy	Yes	411	Ref		Ref	
	No	440	1.42 (1.13–1.790)	**0.003**	0.85 (0.68–1.06)	0.14
Histologic Grade	1	125	Ref		Ref	
	2	464	1.84 (1.26–2.69)	**0.002**	1.42(1.00–2.02)	**0.05**
	3-4	199	2.35 (1.57–3.53)	**< 0.0001**	1.88 (1.29–2.76)	**0.001**
Stage	1	149	Ref		Ref	
	2	293	2.14 (1.32–3.48)	**0.002**	2.47 (1.51–4.05)	**0.0003**
	3	244	3.75 (2.33–6.03)	**< 0.0001**	3.87 (2.38–6.31)	**< 0.0001**
	4	177	11.87 (7.44–18.95)	**< 0.0001**	11.86 (7.42–18.98)	**< 0.0001**

* Ex-smokers included in non-smokers

Abbreviations: HR, hazard ratio; 95% CI, confidence interval. Significant results in bold.

a Numbers may not add up to 100% of available subjects because of missing information

**Figure 1:**
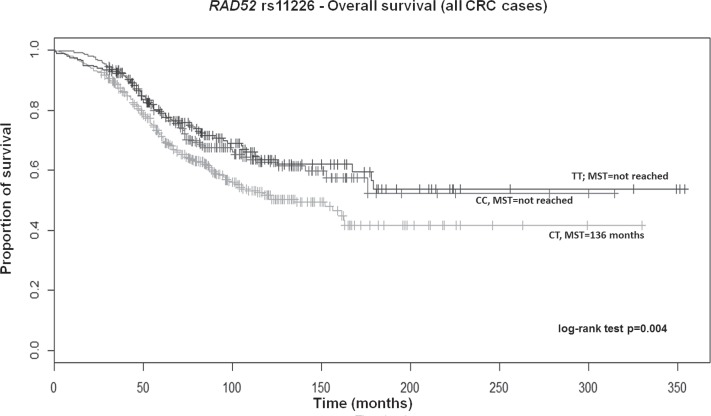
Kaplan-Meier OS curves for *RAD52* rs11226 in all CRC patients MST = median survival time.

**Figure 2:**
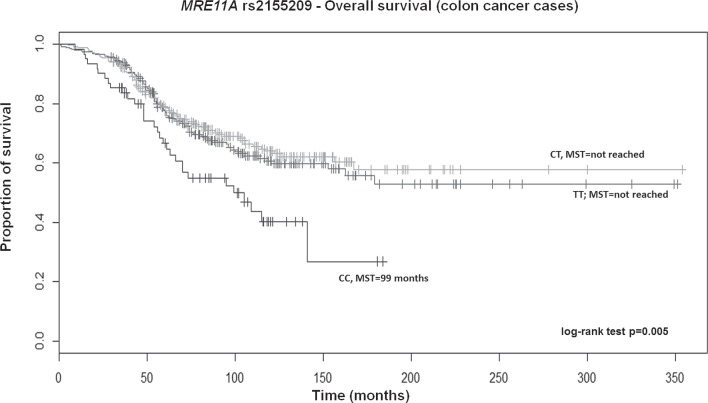
Kaplan-Meier OS curves for *MRE11A* rs2155209 in colon cancer patients MST = median survival time.

After adjusting for above significant covariates, CRC patients, particularly those with colon cancer carrying the TT genotype of *RAD52* rs11226, displayed a longer survival in a recessive model (HR 0.70; 95% CI 0.52–0.93; *p* = 0.02 and HR 0.60; 95% CI 0.41–0.89; *p* = 0.01, respectively; Supplementary Table 4). Overall, patients also showed a similar significant trend across genotypes in the Kaplan–Meier curves (log-rank test *p* = 0.004; Median survival time (MST) for CT carriers was 136 months; MST not reached for the other genotypes; Figure 1). Likewise, a similar trend was also found for colon cancer patients (log-rank test *p* = 0.005; MST for CT carriers was 162 months; MST not reached for the other genotypes; data not shown). Colon cancer patients with the variant CC genotype of *MRE11A* rs2155209 showed a shorter survival when compared with the most frequent TT genotype (HR 1.63; 95% CI 1.06–2.51; *p* = 0.03) or with T-allele carriers (HR 1.54; 95% CI 1.03–2.31; *p* = 0.04) (Supplementary Table 4). A similar trend was observed in the univariate Cox model and in the relative Kaplan–Meier curves (log-rank test *p* = 0.005; MST for CC carriers being 99 months; MST not reached for the other genotypes; Figure 2). No significant association with recurrence risk was observed for any of the HRe genes (Supplementary Table 5).

Overall, no strong associations with survival and risk of recurrence were observed for all analysed miRSNPs in NHEJ genes (Supplementary Table 6 and Supplementary Table 7). Among CRC cases, carriers of the GG genotype of *XRCC5* rs1051685 showed a decreased survival (OS: HR 2.12; 95% CI 1.04–4.32; *p* = 0.04). A similar trend was observed in the univariate Kaplan–Meier curves, although not being statistically significant (log-rank test *p* = 0.07; MST for AA and AG carriers = 176 and 178 months, respectively; MST for GG carriers = 65 months).

### Luciferase assay

**Figure 3:**
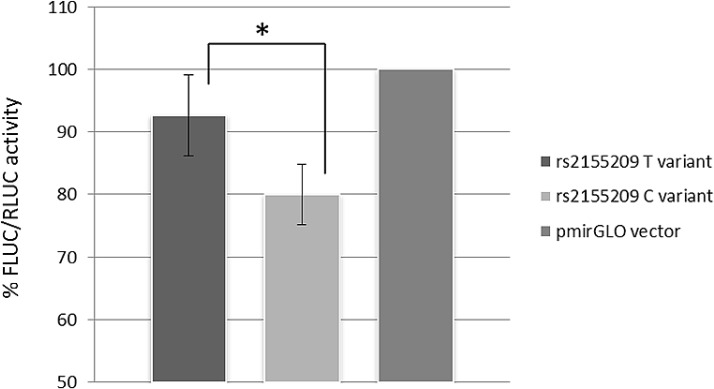
Data show mean values of luminescence activity, normalized to Renilla luciferase levels, (FLUC/RLUC) from four independent experiments MRE11A expression show a statistical significant (*p* = 0.007) decrease of about 14% in presence of the rs2155209 C-variant, compared to the expression obtained with the T-variant.

The role of rs2155209 in modulating *MRE11A* expression was investigated by a dual 3′UTR luciferase reporter assay. A statistically significant difference between the two constructs carrying the different alleles of the SNP was observed (*p* = 0.007, MANOVA). Figure 3 shows the luciferase activity following transfection with the pmirGLO vector without the 3′UTR (used as reference and set as 100%) and with the vectors carrying the alternative alleles in HCT-116 cell line. The average luciferase activity of the vector carrying the C-allele showed a reduction by 14% in comparison with the values obtained for the construct with the T-allele.

### Validation on TCGA database

RNA sequencing (RNAseq) data of CRC patients in The Cancer Genome Atlas (TCGA) database were downloaded. The results from RNAseq from 327 tumor tissues and 13 normal-appearing, adjacent mucosa were available [24]. A general overexpression of all 20 transcripts of *MRE11A* was observed in the tumor tissues when compared with healthy tissues (for all *p* < 10^−7^).

## DISCUSSION

In the present study, we investigated the role of 21 miRSNPs in DSB repair genes in modulating CRC susceptibility and clinical outcome. The major finding was the association of the variant CC genotype of *MRE11A* rs2155209 with a decreased risk of CRC. This association was observed independently of the stratification of the cases according to tumor site recorded at diagnosis. The C-allele of the SNP was also related to a reduced activity of the reporter gene in a dual luciferase assay.

*MRE11A* encodes for a protein that is a component of the MRE11-RAD50-NBS1 (MRN) complex involved in DSB repair by both HRe and NHEJ, in the maintenance of telomere integrity, in DNA recombination during meiosis, and in the signaling of DSB damage [25]. Mutations in *NBS1*, *MRE11A*, and *RAD50* disrupting the functionality of MRN complex may lead to genome instability and carcinogenesis. For instance, these mutations have been reported for acute lymphoblastic leukemia [26], head and neck [27], prostate, breast and colorectal [28] cancers. *MRE11A* has been identified as a possible candidate for breast cancer susceptibility by Bartkova and colleagues [29]. Interestingly, *MRE11* overexpression, commonly observed among cancer patients, has been postulated as a mechanism responsible for increasing cancer risk [24]. To support this hypothesis, RNAseq data available in TCGA database also show an overexpression of all available *MRE11A* transcripts in CRC tumor tissues when compared with their healthy tissue counterparts. In this sense, we may hypothesize that a miRNA post-transcriptional regulation of *MRE11A* may be finely modulated by the presence of the identified miRSNP, with the CC genotype contributing to a reduced risk of developing CRC. The low-risk allele (C) is in fact associated with a lower expression of *MRE11A* most probably due to the C-allele stronger interactions with the putative binding miRNAs, as suggested by the results of the functional study.

To correctly interpret these results, we should not exclude the possibility that the observed association may be due to the *linkage disequilibrium* (LD) structure of the investigated locus. Rs2155209 in the Caucasian population is in a LD block spanning over 125Kbp and containing 31 SNPs. Among those SNPs, it is the only one in the 3′UTR and is indicated as one of the variants in the block describing one of the most common haplotypes (with a frequency of approx 25.4% of haplotypes harbouring the C allele) by Haploview software (HG19). The *MRE11A* 3′UTR hosts several binding sites for transcription factors (for instance SMC3, CTCF, RAD21). However, the region surrounding rs2155209, and including the seed of the miRNAs predicted to bind on the SNP of interest, is not a site for RNA-binding proteins (RBPs), supporting the hypothesis of a miRNA related post-transcriptional mechanism. Although evidence highlighted a potential miRNA-dependent regulation of the gene, *MRE11A* expression could not be affected solely by rs2155209; other SNPs could be causally linked to the risk of CRC by different mechanisms.

A significant role for genetic factors in CRC has been confirmed by genome-wide association studies (GWAS) and large-scale replication studies, which have identified so far 124 SNPs associated with this cancer (the GWAS catalog: https://www.ebi.ac.uk/gwas/search?query=colorectal%20cancer). However, the loci identified were estimated to account collectively for approximately 6% of the excess familial risk of CRC [30], suggesting that additional SNPs remain to be identified. The rs2155209 polymorphism has also been previously associated with an increased risk of myocardial infarction, breast and bladder cancer [31–33]. For the latter, the rare allele was found at an increased risk, but genotype distribution in controls was not found in HW equilibrium. To the best of our knowledge, no reports have documented any association with CRC risk. Interestingly, other genetic variants in *MRE11* (not in linkage with rs2155209) have been associated with various cancers including breast, bladder and ovarium [28, 29, 34, 35].

In the last years, the interest on miRNAs has increased since they have been recognized as pivotal players in diverse biologic processes, including DNA repair and DNA damage response [36, 37]. An increasing body of evidence indicates the possibility to use miRNAs as diagnostic, prognostic and predictive clinical biomarkers [20]. In this context, the presence of SNPs within the 3′UTRs of target DNA repair genes could alter the binding with specific miRNAs, modulating gene expression and ultimately affecting, besides cancer susceptibility [18, 22, 38], also therapy outcomes [39] and survival [21]. As an example, an association between a miRNA binding site SNP within the DNA repair gene *RAD51* with bladder cancer risk and radiotherapy outcomes has been reported [39].

miRNAs typically mediate fine regulation of gene expression, tuning rather than altering protein levels [37]. There is evidence that miRNAs can control DNA damage response by interacting with DNA repair genes. Most of the studies have been conducted on cancer cell lines, and it is not clear whether miRNAs mediate DNA repair in healthy cells [37]. Most recently it has been hypothesized that high expression levels of DNA repair proteins are detrimental to DSB repair as the stoichiometry of factors in specific pathways is important. miRNAs could then facilitate DNA repair by maintaining the optimal levels of repair proteins [37], and there could be a further modulation mediated by SNPs in miRNA seeds or in target regions. In the context of CRC and DNA repair, our group has provided the first evidence that variations in miRNA-binding sites in Base Excision Repair genes 3′UTRs may modulate prognosis and therapy response [21]. In the present study, among CRC patients, and specifically those with colon cancer, carriers of the TT genotype of *RAD52* rs11226 displayed a better survival while carriers of the *MRE11A* rs2155209 variant CC genotype showed a shorter survival. Notably, MRE11 protein deficiency has been recently observed to be associated with improved survival of stage III colon cancer patients, independently of treatment [40]. This study supports our finding where CC genotype of *MRE11A* rs2155209 is associated with shorter survival. We can theorize that the modulatory role by the observed SNP on the expression of MRE11 protein may also influence the prognosis of cancer. RAD52 is a key protein in the homologous recombination pathway. In humans, it is known to exist in an oligomeric form in order to bind single-stranded DNA (ssDNA), to promote ssDNA annealing, to interact with RPA, and under certain specialized conditions, to simulate Rad51-mediated homologous DNA pairing [41]. There is an established interplay between *MRE11A* and *RAD52* genes since the binding of MRN complex to a DSB permits a following recruitment of RAD52 to start the resolution of the damage [42]. Both genes have numerous predicted binding miRNAs in their 3′UTRs, although only very few of them have been validated so far. We have investigated miRNAs predicted to bind to *RAD52* and *MRE11A* where the SNPs found in association lie (reported in Supplementary Table 8). From the available data (source http://www.genecards.org/), many of these miRNAs are expressed in colon tissue. Interestingly, among them, two miRNAs (miR-1296 and miR-296–5p) are predicted to bind both genes. These miRNAs have been described deregulated in cancer and other diseases and, in particular, miR-296–5p has frequently been associated with cancer prognosis [43, 44].

Protein expression levels of NBS1, MRE11, and RAD50 in malignant tissues have also been measured in previous studies. For instance, it was observed that a lower MRE11 expression in tumor cells in bladder and breast tissues was also associated with worse cancer-specific survival compared with high expression [45, 46], and the underlying control mechanism determining these lower expression levels was essentially post-transcriptional and regulated by miR-153 [47]. Additionally, *RAD50* and *NBS1* mRNA levels correlated with expression of all three proteins, implying that transcription of these two genes determines the amount of MRN complex formed. In this sense, MRE11 protein levels seem to adapt in line with the complex formation, with the following degradation of protein molecules that are not required for complex formation [47]. This strong interconnection may explain the other observed associations, in particular for the variants related to patient clinical outcome.

To our knowledge, this is the first study comprehensively investigating the role of SNPs residing in miRNA target sites of DSB repair genes in association with CRC risk and clinical outcome. The study population included in the present work is genetically homogeneous (all Caucasian from the Czech Republic), and clinically well-defined (cases and controls recruited in the same centers with follow-up data collected by the same physicians), thus excluding any possible population stratifications and bias. In addition, the inclusion of ‘colonoscopically negative’ individuals ensured disease-free control individuals because a negative colonoscopy result is the best available proof of the CRC absence [48]. Since this group of individuals may not necessarily represent the general population, we also included healthy cancer-free individuals recruited among volunteers from blood centers.

We are aware of certain limitations of the present investigation. In the case-control study, controls differed from cases in age and gender distribution, as well as other parameters such as BMI. However, we attempted to control tentative age effect by matching cases and controls by age quartiles through bootstrap sampling, and no changes were observed in the ten different resamplings.

The main and novel finding of the present study was that *MRE11A* rs2155209 resulted strongly associated with a decreased risk of CRC, taking into account also multiple comparisons (by considering a 5% Bonferroni-corrected significance threshold). Moreover, the presence of one or the other allele of rs2155209 was associated with a different luciferase activity. The present results support the emerging idea of a “miRNA network” that may contribute to CRC [49]. Other miRSNPs, both in the same gene and in other DSB repair genes, were also associated with clinical outcomes highlighting the importance of this repair pathway in survival, most probably as a consequence of an impaired DNA repair system.

It is generally accepted that all DNA repair pathways act in an integrative and collaborative way. Numerous factors affect the decision to repair a DSB via NHEJ or HRe, and accumulating evidence suggests these major repair pathways both cooperate and compete with each other at DSB sites to facilitate efficient repair and promote genomic integrity [50, 51]. We have observed in particular that both *MRE11A* and *RAD52* share miRNAs predicted to bind to regions where SNPs were associated with survival while a SNP interaction analyses revealed an under-representation of certain genotypes among concomitant genotypes of SNPs in both genes in association with CRC risk. However, a larger population is necessary to test the interaction/cooperation of different genes/SNPs in various pathways.

In conclusion, we identified plausible candidate miRSNPs potentially affecting miRNA binding in DSB repair genes that were related either to CRC susceptibility or to patients’ survival. Further studies are needed to replicate our findings and assess these miRSNPs as predictive biomarkers in independent populations, to functionally characterize the significant genetic variants and to find the biologic mechanisms underlying the associations.

## MATERIALS AND METHODS

### Study population and data collection

Blood samples were collected from 1126 patients with histologically confirmed CRC attending between September 2003 and October 2010 several oncological departments in the Czech Republic (three in Prague, one in Benesov, Brno, Liberec, Ples, Pribram, Usti nad Labem, and Zlin). Two control groups, whose samples were collected at the same time of cases recruitment, were included in the study. The first group consisted of 688 hospital-based individuals admitted to five of the above mentioned gastroenterological departments that had negative colonoscopy results for malignancy or idiopathic bowel diseases (Control Group 1). The reasons for undergoing the colonoscopy were: i) positive fecal occult blood test, ii) hemorrhoids, iii) abdominal pain of unknown origin, and iv) macroscopic bleeding. The second group of controls consisted of 781 healthy blood donor volunteers (Control Group 2) collected from a blood donor centre in Prague. All individuals were subjected to standard examinations to verify the health status for blood donation and were cancer-free at the time of the sampling. Among the CRC cases, 397 patients were diagnosed with a tumor in the colon, 334 in the sigmoideum and 377 with rectal cancer (3 cases were lacking the information about the site of the tumor; however, since they had complete survival data, they remained in the survival analysis). Out of the 1469 controls, 688 were cancer-free colonoscopy inspected controls (Control Group 1) and 781 were healthy blood donor volunteers (Control Group 2). Details of CRC cases and controls have been reported previously [21].

All subjects were informed and provided written consent to participate in the study and to approve the use of their biological samples for genetic analyses, according to the Helsinki declaration. The design of the study was approved by the local Ethics Committee. Study subjects provided information on their lifestyle habits, BMI, diabetes, and family/personal history of cancer, using a structured questionnaire to determine demographic characteristics and potential risk factors for CRC.

### Follow-up of patients

Eight hundred sixty-six CRC cases were monitored with follow-ups until August 31st, 2011. A second group consisting of 232 CRC patients was recruited later on and followed up until March 31st, 2013. For all subjects, clinical data at the time of diagnosis, including location of the tumor, UICC (International Union Against Cancer) tumor-node-metastasis (TNM) stage system, grade and adjuvant chemotherapy treatment were collected, along with information about distant metastasis, relapse and date of death [52].

Four hundred and eleven CRC cases received a 5-FU-based adjuvant regimen as first-line postoperative therapy. The therapy consisted of either a Mayo regimen, delivered as a bolus infusion of 5-FU (425 mg/m^2^) and leucovorin (10 mg/m^2^) for five days every four weeks six times or a simplified De Gramont regimen which consisted of a 2 h intravenous (i.v.) infusion of leucovorin (200 mg/m^2^), then a 5-FU i.v. bolus (400 mg/m^2^) followed by a 46 h 5-FU continuous i.v. infusion (2400–3000 mg/m^2^). Four hundred forty subjects did not receive any adjuvant chemotherapy after surgery. In this study, the outcome variables measured were 5-FU-based chemotherapy, OS (time from diagnosis until death or censorship), and EFS (time of surgery or end of chemotherapy until date of relapse, death or censorship).

### Selection of candidate genes and SNPs in miRNA target binding sites

From the complete list of DNA repair genes available online (http://sciencepark.mdanderson.org/labs/wood/DNA_Repair_Genes.html March 2014 version), seven genes were retrieved in the NHEJ pathway and 21 genes in the HRe pathway.

The approach used to select the candidate miRSNPs was similar to that described in [21]. Briefly, for each gene, SNPs within target binding sites for miRNAs were identified by using the freely available software: *MicroSNiper* (http://cbdb.nimh.nih.gov/microsniper [53], *MiRSNP* (http://202.38.126.151/hmdd/mirsnp/search/ [54]), *Mirnsnpscore* (http://www.bigr.medisin.ntnu.no/mirsnpscore/ [55]), and *Polymirt* (http://compbio.uthsc.edu/miRSNP/ [56]). The 50 detected SNPs were then filtered for their minor allele frequency (MAF > 5%) in Caucasian populations in the SNP database to reach an appropriate representation of all genotypes in our set of cases and controls. The information was primarily derived from 1000genomes project database, phase 1, CEU population; whenever this was not possible, other reference populations were considered (i.e. HAPMAP CEU population) (dbSNP; http://www.ncbi.nlm.nih.gov/SNP/). SNPs with the required MAF were further tested for the possibility to be in LD using HaploView (v. 4.2) with the data from HapMap v. 3, release R2 in the CEU population.

### SNP genotyping

Genomic DNA was isolated from peripheral blood lymphocytes using standard procedures. The DNA from cases and controls was randomly placed on plates where an equal number of samples could be run simultaneously. The selected SNPs were genotyped using the KASP^™^ genotyping assay, a competitive allele-specific PCR SNP genotyping system (LGC Genomics, Hoddesdon, Herts, UK). For quality control purposes, duplicate samples (5% of the total numbers of samples) were repeated for each SNP, no template controls were included in each plate (NTCs).

### DNA cloning and *in vitro* assay

A Dual-Luciferase reporter assay was used to investigate whether the *MRE11A* rs2155209 alleles were associated with a differential gene expression. Initially, a 1031 bp fragment of the 3′UTR region of *MRE11A* containing the T-allele of the SNP was PCR-amplified. The PCR primers were specifically designed to allow the cloning reaction with ClonEZ enzyme. The bases at the primers 3′ ends were specific to the region to be amplified, whereas the 15 bases at the 5′ ends were homologous to either side of the *XhoI* restriction site within the multiple cloning sites of the pmirGLO vector (Promega, Madison, USA). Each primer was also designed to include a *XhoI* restriction site sequence (c^tcgag) between the two sequences. The complete sequences were: sense primer = AACGAGCTCGCTAGCCTCGAGGGGTGATAAATCTCTCCAGCTAATTC; and anti-sense primer = CAGGTCGACTCTAGACTCGAGAGCCCATTGAGATACTTTTTTACTCAG. The vector was linearized with *XhoI* (NEB Inc, Ipswich, USA) and the PCR product was cloned downstream from the firefly luciferase (*Photinus pyralis*) reporter gene, using the Clone EZ PCR Cloning Kit (Genscript, Piscataway, USA). Competent cells NZY5α (NZYTech, Lisbon, Portugal) were used for transformation after the cloning reaction, as suggested by manufacturers. To obtain a vector with an *MRE11A* 3′UTR bearing the C-allele of rs2155209, the construct underwent site-specific mutagenesis using the Quick Change Lightning Site Direct Mutagenesis kit (Agilent, Milano, Italy). The sequences of the mutagenic primers were: sense = attgttttctcctttctgggtaacacgccctaacttctg; and anti-sense = cagaagttagggcgtgttacccagaaaggagaaaacaat. Following the digestion of the parental (methylated) supercoiled double-stranded DNA with *Dpn I*, *XL10-Gold* ultra-competent cells (Agilent, Milano, Italy) were used for transformation.

For the functional assay, HCT-116 cells were plated at a density of approximately 7 × 10^4^ cells per well in 24-well plates and incubated overnight at 5% CO_2_, 37°C in a humidified incubator. Cells were transiently transfected at about 80% confluence using 3 μl of Polyfect transfection reagent (Qiagen, Milano, Italy) and 0.4 μg of the chimeric construct carrying the T or the C allele.

The assays were carried out using the dual-luciferase reporter assay kit (Promega, Milano, Italy). A pmirGLO vector without 3′UTR insert was used as a reference. PmirGLO vectors contain the luciferase gene from *Renilla reniformis* (hRluc-neo), acting as a control reporter to normalize transfection efficiency. Twenty-four hours after transfection, cells were washed with a phosphate-buffered saline solution and lysed with 100 μl of Passive Lysis Buffer (PLB Promega, Milano, Italy) for an optimal stability of the firefly and *Renilla* luciferase reporter enzymes. The culture vessel was shaken for 8 minutes at room temperature. The lysates were used for measuring the activity of firefly (FLUC) and *Renilla* (RLUC) luciferases. Three replicates of all experimental points were performed in each experiment. For each transfection, luminescence intensity was evaluated by a luminometer (Optima FluoStar, BMG, Ortenberg, Germany), and luciferase activities were averaged from four measurements. The luminescence intensities of firefly and *Renilla* luciferase of the non-transfected cells (background) were subtracted from the values obtained for the transfected cells with the pmiRGLO vector containing the 3′UTR. The luminescence of the *Renilla* luciferase was used as the control reporter to calculate the normalized firefly luciferase activity (FLUC/RLUC activity).

### Statistical analyses

Pearson's chi-square test (1 degree of freedom), with a type-I error threshold set at α = 0.05, was used to verify whether the genotypes were in Hardy-Weinberg equilibrium in controls. SNPs were excluded from further analyses if the call rate was < 95%, deviated from Hardy-Weinberg equilibrium in controls at *p* < 10^2^, or if genotypes were discrepant in more than 2% of duplicate samples. The multivariate logistic regression (MLR) analysis was used to test the association between genotypes and risk of CRC. The covariates analysed in the multivariate model were: sex, age, smoking habit (non-smokers vs. smokers and ex-smokers), BMI, familial history of CRC, education level (high, intermediate and low) and living area (country, suburbs, and town). The associations between SNPs and CRC risk were calculated by estimating the ORs and their 95% CI, adjusted for both continuous and discrete covariates. For all the genotypes, regression coefficients for additive models were estimated. For each SNP, we evaluated its association with cancer risk using two different genetic models—dominant, and recessive—to define the best fitting model with the most significant *p*-value. The Bonferroni-corrected significance threshold is 0.002 (for 21 SNPs and α = 0.05).

The model with the highest likelihood was additionally checked for the significance of possible interaction terms in the MLR analysis. Statistical analyses were performed using R (http://www.rproject.org).

OS in CRC patients was estimated using the date of death or the date of follow-up termination as the end point. For the EFS, in patients who did not have distant metastasis at the time of diagnosis, date of relapse, death or end of the study were used as the end point of follow-up. EFS was defined as the time from surgery/end of therapy to the occurrence of distant metastasis, recurrence or death, whichever came first. The survival curves for OS and EFS were derived by the Kaplan–Meier method (R version 2.14–2, Survival package). The relative risk of death was estimated as HR using Cox regression (R version 2.14–2, Survival package). Multivariate survival analyses were adjusted for age, gender, smoking and stage.

For the *in vitro* assays, the ratios (FLUC/RLUC) of the measurements of luminescence, each subtracted of its respective background, were compared between genotypes using the multifactor analysis of variance with interactions (MANOVA), where “experiment” and “genotype” were entered as independent factors in the model. The statistical tests were 2-tailed and carried out using Statgraphics Centurion software (StatPoint Technologies, Warrenton, Va).

## SUPPLEMENTARY MATERIALS TABLES




