# Supplementary material for: Double-strand break repair and colorectal cancer: gene variants within 3′ UTRs and microRNAs binding as modulators of cancer risk and clinical outcome
Source: Oncotarget. 2015 Dec 31;7(17):23156–69. doi: 10.18632/oncotarget.6804 (PMC5029617; doi:10.18632/oncotarget.6804)
Supplement: Supplementary file 1 [file oncotarget-07-23156-s001.pdf]

Double-strand break repair and colorectal cancer: gene variants within 3′ UTRs and microRNAs binding as modulators of cancer risk and clinical outcome

Supplementary Materials

Supplementary Table SI: Association of SNPs in HRe genes with CRC risk. A further stratification for colon and rectal cancer is also presented

Supplementary Table SII: Association of SNPs in NHEJ genes with CRC risk. A further stratification for tumor site is also presented

Supplementary Table SIII: Combined 2 × 2 contingency tables showing the distribution of rs2155209 genotypes of *MRE11A* with that of other SNPs in genes of the HRe pathway: only the relevant associations are reported

		***MRE11A* rs2155209 (Cases/Controls)**		
	**Genotype**	**TT**	**TC**	**CC**
RAD52 **rs1051669**	GG	(285/344)<br>OR = 1.13, $\chi^2 = 1.67$,<br>$p = 0.20$	(269/376)<br>OR = 0.93, $\chi^2 = 0.70$,<br>$p = 0.40$	(43/103)<br>OR = 0.53, χ2 = 11.81,<br>**$p < 0.001$**
	GA	(170/221)<br>OR = 1.02, $\chi^2 = 0.03$,<br>$p = 0.87$	(169/211)<br>OR = 1.07, $\chi^2 = 0.34$,<br>$p = 0.56$	(39/67)<br>OR = 0.76, χ2 = 1.80,<br>$p = 0.18$
	AA	(31/29)<br>OR = 1.42, $\chi^2 = 1.84$,<br>$p = 0.18$	(41/33)<br>OR = 1.67, $\chi^2 = 4.71$,<br>**$p = 0.03$**	(7/7)<br>OR = 1.32, χ2 = 0.27,<br>$p = 0.60$
RAD52 **rs11571475**	TT	(369/442<br>OR = 1.16), $\chi^2 = 2.99$,<br>$p = 0.08$	(354/458)<br>OR = 1.03, $\chi^2 = 0.15$,<br>$p = 0.69$	(81/126)<br>OR = 0.84, χ2 = 1.41,<br>$p = 0.24$
	TC	(114/151)<br>OR = 1, $\chi^2 = 0$,<br>$p = 0.99$	(109/152)<br>OR = 0.94, χ2=0.19,<br>$p = 0.66$	(10/46)<br>OR = 0.28, χ2 = 14.82,<br>**$p < 0.001$**
	CC	(11/9)<br>OR = 1.62, $\chi^2 = 1.18$,<br>$p = 0.28$	(8/11)<br>OR = 0.96, $\chi^2 = 0.01$,<br>$p = 0.93$	(1/4)<br>OR = 0.33, χ2 = 1.08,<br>$p = 0.30$
GEN1 **rs300171**	CC	(191/231)<br>OR = 1.10, $\chi^2 = 0.82$,<br>$p = 0.36$	(179/258)<br>OR = 0.89, $\chi^2 = 1.16$,<br>$p = 0.28$	(47/69)<br>OR = 0.88, χ2 = 0.40,<br>$p = 0.53$
	CT	(206/263)<br>OR=1.03, $\chi^2 = 0.10$,<br>$p = 0.75$	(228/271)<br>OR = 1.13, $\chi^2 = 1.50$,<br>$p = 0.22$	(32/82)<br>OR = 0.50, χ2 = 11.27,<br>**$p < 0.001$**
	TT	(87/88)<br>OR = 1.32, $\chi^2 = 3.19$,<br>$p = 0.07$	(62/82)<br>OR = 0.99, $\chi^2 = 0.00$,<br>$p = 0.96$	(10/22)<br>OR = 0.59, χ2 = 1.91,<br>$p = 0.17$
XRCC2 **rs3218547**	CC	(299/352)<br>OR = 1.18, $\chi^2 = 3.32$,<br>$p = 0.07$	(283/369)<br>OR = 1.03, $\chi^2 = 0.09$,<br>$p = 0.76$	(48/108)<br>OR = 0.57, χ2 = 10.00,<br>**$p < 0.001$**

	CA	(169/224) OR = 1.01, $\chi^2 = 0.00$, $p = 0.96$	(176/218) OR = 1.09, $\chi^2 = 0.60$, $p = 0.44$	(37/59) OR = 0.83, χ2 = 0.77, $p = 0.38$
	AA	(18/27) OR = 0.89, $\chi^2 = 0.16$, $p = 0.69$	(22/39) OR = 0.75, $\chi^2 = 1.19$, $p = 0.27$	(5/11) OR = 0.60, χ2 = 0.89, $p = 0.34$

OR = odds ratio. Significant results in bold.

Supplementary Table SIV: SNPs in HRe genes associated with OS in the whole CRC cases and stratified for tumor site (Cox regression for adjusted estimates)

Supplementary Table SV: SNPs in HRe genes associated with EFS in the whole CRC cases and stratified for tumor site (Cox regression for adjusted estimates)

Supplementary Table SVI: SNPs in NHEJ genes associated with OS in the whole CRC cases and stratified for tumor site (Cox regression for adjusted estimates)

Supplementary Table SVII: SNPs in NHEJ genes associated with EFS in the whole CRC cases and stratified for tumor site (Cox regression for adjusted estimates)

Supplementary Table SVIII: miRNAs predicted to bind in the region surrounding *MRE11A* rs2155209 and *RAD52* rs11226 SNPs by different tools

Gene and polymorphism	Tool for SNP prediction miRNAs	miRNAs shared by different tools for prediction	miRNA entry in miRBase	Expression in colon (Genecards)[1]
MRE11A rs2155209	**microSNIPer (http://cbdb.nimh.nih.gov/microsniper)**			
	hsa-miR-1234-3p		MIMAT0005589	Expressed in colon (microarray)
	hsa-miR-1258		MIMAT0005909	No Expression Related Data Available
	hsa-miR-1267		MIMAT0005921	Scarcely expressed in colon (RNAseq)
	**hsa-miR-1296**	X	MIMAT0005794	Expressed in colon and sigmoideum (RNAseq)
	hsa-miR-26b-3p		MIMAT0004500	Scarcely expressed in colon (RNAseq)
	hsa-miR-27a-5p	X	MIMAT0004501	Expressed in colon transverse (less in sigmoideum) (RNAseq)
	hsa-miR-2909		MIMAT0013863	No Expression Related Data Available
	**hsa-miR-296-5p**		MIMAT0000690	Expressed in colon (sigmoideum, transverse) (RNAseq)
	hsa-miR-3158-3p	X	MIMAT0015032	Expressed in colon transverse (less in sigmoideum) (RNAseq)
	hsa-miR-3655		MIMAT0018075	Highly expressed in colon (sigmoideum, transverse) (RNAseq)
	hsa-miR-4446-3p		MIMAT0018965	No Expression Related Data Available

	hsa-miR-4512		MIMAT0019049	Expressed in colon transverse (less in sigmoideum) (RNAseq)
	hsa-miR-4726-5p		MIMAT0019845	Scarcely expressed in colon (RNAseq)
	hsa-miR-5001-5p		MIMAT0021021	Highly expressed in colon (sigmoideum, transverse) (RNAseq)
	hsa-miR-5683		MIMAT0022472	No Expression Related Data Available
	hsa-miR-600		MIMAT0003268	Expressed in colon (microarray)
	hsa-miR-6069		MIMAT0023694	No Expression Related Data Available
	hsa-miR-6514-3p		MIMAT0025485	No Expression Related Data Available
	**Polymirt (http://compbio.uthsc.edu/miRSNP)**			
	hsa-miR-5088-5p			Scarcely expressed in colon (RNAseq)
	hsa-miR-6895			No Expression Related Data Available
	hsa-miR-7160-3p			No Expression Related Data Available
	**Mirnsnpscore (http://www.bigr.medisin.ntnu.no/mirsnpscore/)**			
	hsa-miR-3158 (now hsa-miR-3158-3p)	X		Expressed in colon transverse (less in sigmoideum) (RNAseq)
	hsa-miR-2861			No Expression Related Data Available
	**hsa-miR-1296**	X		Expressed in colon and sigmoideum (RNAseq)
	hsa-miR-27a* (now hsa-miR-27a-5p)	X		Expressed in colon transverse (less in sigmoideum) (RNAseq)
	hsa-miR-214* (hsa-miR-214-5p)			Highly expressed in colon (sigmoideum, transverse) (RNAseq)
RAD52 rs11226	**microSNIPer (http://cbdb.nimh.nih.gov/microsniper)**			
	**hsa-miR-296-5p**		MIMAT0000690	Expressed in colon (sigmoideum, transverse) (RNAseq)
	hsa-miR-3614-5p		MIMAT0017992	Highly expressed in colon (sigmoideum, transverse) (RNAseq)
	hsa-miR-4260		MIMAT0016881	Highly expressed in colon (sigmoideum, transverse) (RNAseq)
	hsa-miR-496		MIMAT0002818	Moderately expressed in colon transverse (scarcely in sigmoideum) (RNAseq)
	hsa-miR-541-3p		MIMAT0004920	Moderately expressed in colon transverse (scarcely in sigmoideum) (RNAseq)
	hsa-miR-1225-3p		MIMAT0005573	Expressed in colon and sigmoideum (RNAseq)
	**hsa-miR-1296**		MIMAT0005794	Expressed in colon and sigmoideum (RNAseq)
	hsa-miR-3136-3p	X	MIMAT0019203	Scarcely expressed in colon (RNAseq)
	hsa-miR-4690-3p		MIMAT0019780	Scarcely expressed in colon (RNAseq)
	hsa-miR-890		MIMAT0004912	Scarcely expressed in colon (RNAseq)
	hsa-miR-1233-1-5p		MIMAT0022943	No Expression Related Data Available
	hsa-miR-1233-3p		MIMAT0005588	No Expression Related Data Available

	hsa-miR-1587		MIMAT0019077	No Expression Related Data Available
	hsa-miR-3147		MIMAT0015019	No Expression Related Data Available
	hsa-miR-3151		MIMAT0015024	No Expression Related Data Available
	hsa-miR-3189-3p		MIMAT0015071	No Expression Related Data Available
	hsa-miR-5591-5p		MIMAT0022301	No Expression Related Data Available
	hsa-miR-6075		MIMAT0023700	No Expression Related Data Available
	hsa-miR-6724-5p		MIMAT0025856	No Expression Related Data Available
	**Polymirt (http://compbio.uthsc.edu/miRSNP)**			
	hsa-miR-3136-3p	X	MIMAT0019203	Scarcely expressed in colon (RNAseq)
	hsa-miR-635			Highly expressed in colon (sigmoideum,transverse) (RNAseq)

In Italics miRNAs shared by both alleles. In bold miRNAs shared by both SNPs
[1]For RNAseq data retrieved from GTEx, for microarray from BioGPS.
